# Effectiveness and cost-effectiveness of a multicomponent intervention to implement a clinical practice guideline for systemic lupus erythematosus: protocol for a cluster-randomized controlled trial

**DOI:** 10.1186/s12913-019-4589-9

**Published:** 2019-11-01

**Authors:** María M. Trujillo-Martín, Yolanda Ramallo-Fariña, Tasmania del Pino-Sedeño, Íñigo Rúa-Figueroa, Elisa Trujillo-Martín, Laura Vallejo-Torres, Iñaki Imaz-Iglesia, Ricardo Sánchez-de-Madariaga, Ana M. de Pascual-Medina, Pedro Serrano-Aguilar, SLE-CPG-Implementation Group, Pilar García Sagredo, Arantxa Arteaga González, Laura Casas Hernández, Monica Delgado Sanchez, Laura Magdalena Armas, Cristina Rodríguez Regalado, Jose A. Hernández Beriain, Francisco J. Novoa Medina, Beatriz Tejera Segura, Monica Troche Duarte, Celia Erausquin, Cristina Almeida, Esmeralda Delgado Frias, Vanesa Hernández Hernández, Ivan Feraz Amaro, Beatriz Rodriguez Lozano

**Affiliations:** 1 Fundación Canaria Instituto de Investigación Sanitaria de Canarias (FIISC), Santa Cruz de Tenerife, Tenerife, Spain; 2 Red de Investigación en Servicios de Salud en Enfermedades Crónicas (REDISSEC), Madrid, Spain; 3 Red Española de Agencias de Evaluación de Tecnologías Sanitarias (RedETS), Madrid, Spain; 4 Centro de Investigaciones Biomédicas de Canarias (CIBICAN), Santa Cruz de Tenerife, Spain; 5 Servicio de Reumatología, Hospital Universitario de Gran Canaria Dr. Negrin, Las Palmas de Gran Canaria, Spain; 6 Servicio de Reumatología, Hospital Universitario de Canarias, La Laguna, Spain; 7 Departamento de Métodos Cuantitativos en Economía y Gestión, Universidad de Las Palmas de Gran Canaria, Las Palmas de Gran Canaria, Spain; 8 Agencia de Evaluación de Tecnologías Sanitarias del Instituto de Salud Carlos III, Madrid, Spain; 9 Unidad de Investigación en Telemedicina y e-Salud, Instituto de Salud Carlos III, Madrid, Spain; 10 Servicio de Evaluación del Servicio Canario de la Salud (SESCS), Servicio Canario de la Salud, Santa Cruz de Tenerife, Spain

**Keywords:** Care management, Clinical practice guideline, Cost-effectiveness, Decision support aids, Electronic communication, Implementation, Knowledge transfer, Multicomponent intervention, Secondary care, Systemic lupus erythematosus

## Abstract

**Background:**

Systemic lupus erythematosus (SLE) is a heterogeneous autoimmune disease with significant potential morbidity and mortality. Substantial gaps have been documented between the development and dissemination of clinical practice guidelines (CPG) and their implementation in practice. The aim of this study is to assess the effectiveness and cost-effectiveness of a multi-component knowledge transfer intervention to implement a CPG for the management of SLE (CPG-SLE).

**Methods:**

The study is an open, multicentre, controlled trial with random allocation by clusters to intervention or control. Clusters are four public university hospitals of the Canary Islands Health Service where rheumatologists are invited to participate. Patients diagnosed with SLE at least one year prior to recruitment are selected. Rheumatologists in intervention group receive a short educational group programme to both update their knowledge about SLE management according to CPG-SLE recommendations and to acquire knowledge and training on use of the patient-centred approach, a decision support tool embedded in the electronic clinical record and a quarterly feedback report containing information on management of SLE patients. Primary endpoint is change in self-perceived disease activity. Secondary endpoints are adherence of professionals to CPG-SLE recommendations, health-related quality of life, patient perception of their participation in decision making, attitudes of professionals towards shared decision making, knowledge of professionals about SLE and use of healthcare resources. Calculated sample size is 412 patients. Data will be collected from questionnaires and clinical records. Length of follow-up will be 18 months. Multilevel mixed models with repeated time measurements will be used to analyze changes in outcomes over time. Cost-effectiveness, from both social and healthcare services perspectives, will be analyzed by measuring effectiveness in terms of quality-adjusted life years gained. Deterministic and probabilistic sensitivity analyses are planned.

**Discussion:**

Impact of CPGs in clinical practice could be improved by applying proven value interventions to implement them. The results of this ongoing trial are expected to generate important scientifically valid and reproducible information not only on clinical effectiveness but also on cost-effectiveness of a multi-component intervention for implementation of a CPG based on communication technologies for chronic patients in the hospital setting.

**Trial registration:**

ClinicalTrial.gov NCT03537638. Registered on 25 May 2018.

## Background

Systemic lupus erythematosus (SLE) is a chronic, systemic, autoimmune rheumatic disease with large differences in prevalence (0.3–23.2/100,000) depending on geographic context [1]. It is characterized by a wide spectrum of clinical presentations with an unpredictable relapsing-remitting course resulting from its effect on multiple organs. Due to its systemic nature and severity, SLE has potential impact on the physical, psychological and social well-being of people affected [2].

SLE is a prototypic systemic autoimmune disease whose clinical management faces real challenges to diagnose and treat patients, requiring the coordinated action of a variety of medical specialties [3]. However, healthcare for patients with SLE is often fragmented, with significant disparities in SLE management among professionals and specialties [4].

Far from current efforts to promote person centred care, unwarranted medical practice variability and healthcare fragmentation negatively impact health outcomes in SLE patients [5]. Insufficient control of the disease results in an increase in flares, compromising quality of life and productivity of patients and leading to greater use of health services [6], resulting in a considerable burden for patients, caregivers and healthcare systems [7, 8]. All these reasons justify the importance of developing and assessing strategies to guide clinical decisions, favouring integrated and patient centred care to improve patients´ clinical management and outcomes as well as health system sustainability [5, 9].

For this purpose, the Spanish Ministry of Health funded development of the first complete and multidisciplinary clinical practice guideline (CPG) for the management of SLE (CPG-SLE) [10], including patients’ perspective [11]. This evidence-based CPG-SLE is intended to assist practitioner and patient decisions about the most appropriate healthcare for specific clinical circumstances.

Despite major efforts to develop CPGs, there is agreement on the limited impact that these tools are having on professionals’ decisions and patients’ health outcomes [12]. Under-utilization of CPGs has been accounted for lack of resources, lack of time and lack of communication and negotiation skills of healthcare professionals, low self-perceived efficacy in the management of CPGs or limited institutional support for implantation, among other reasons [13]. Therefore, publication of CPGs does not, on its own, automatically results in their use and some kind of implementation strategy is needed [14].

Adherence to CPG recommendations in everyday clinical practice requires attitudinal and behavioural changes among health professionals and a certain adaptation of the structural environment [14–16]. In addition, patient-centredness and shared decision-making are key principles for CPG adherence, accommodating patient priorities and resources [17]. Available evidence indicates that multifaceted CPG implementation strategies can improve adherence to recommendations [13, 16, 18], by combining educational outreach [19], feedback [20] and computerized decision support systems [21, 22], as intervention components, among others.

The aim of this study is to assess the effectiveness and cost-effectiveness of a multi-component intervention of knowledge transfer and decision support for rheumatologists to efficiently enhance the implementation of the systematically developed CPG-SLE and improve patients’ outcomes and health system sustainability.

## Methods

### Trial design

**Fig. 1:**
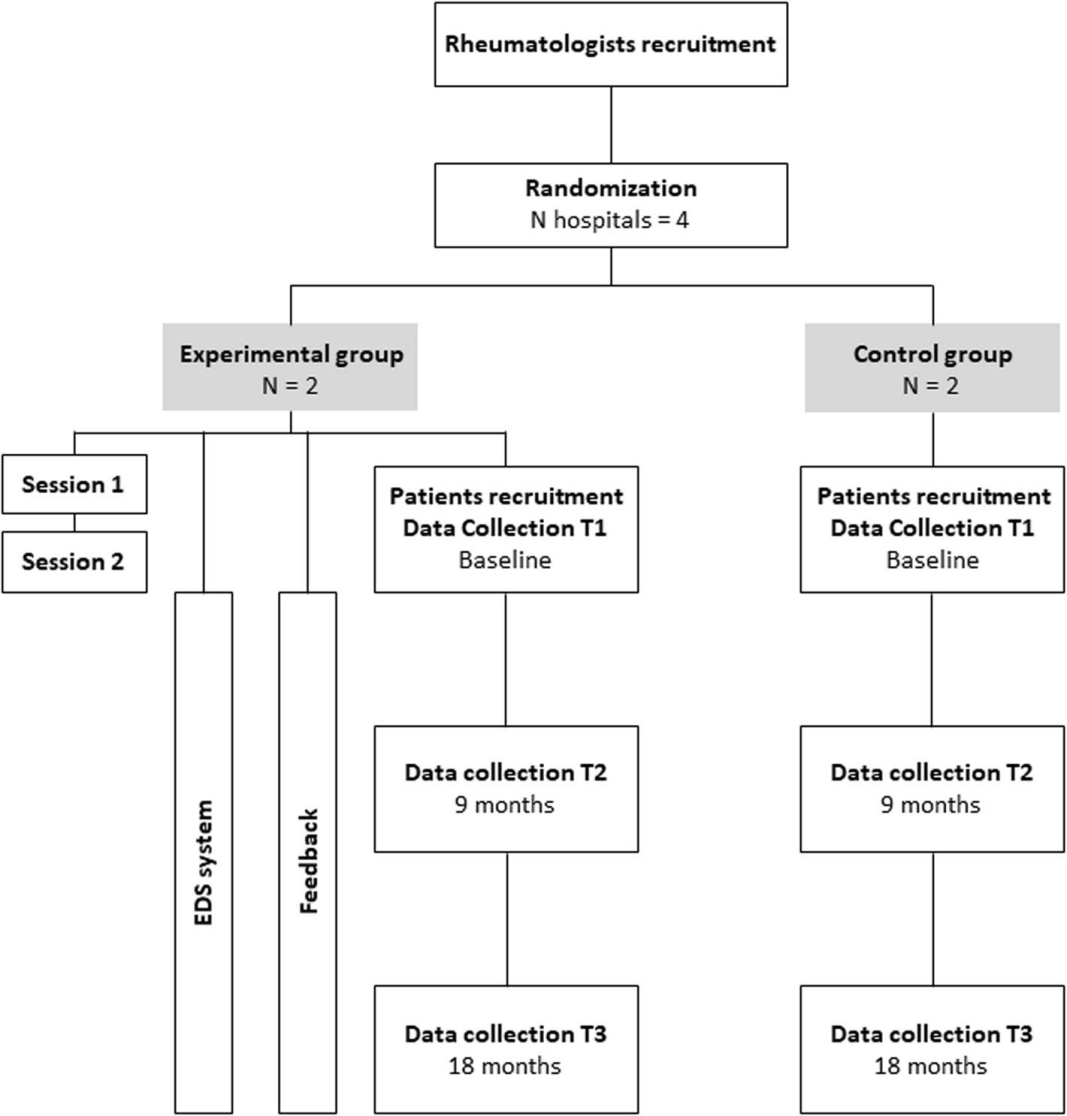
Flowchart of study procedures. EDS: electronic decision support

This is an open, multicentre, two-arm controlled trial with random allocation by clusters (hospitals) to the control arm, where rheumatologists receive standard CPG dissemination procedures provided by the Spanish Healthcare Service (SHS), or to the intervention arm, where rheumatologists receive an experimental three-component intervention in addition to standard CPG dissemination procedures provided by the SHS (Fig. 1).

### Subjects

#### Patients

Even though SLE is a complex multisystemic disease that often requires involvement of different specialists, our intervention for CPG-SLE implementation is limited to rheumatologists. Therefore, to ascertain potential improvement in main outcomes, we selected SLE patients in their initial or mild stages of the disease, which are treated mainly by rheumatologists.

Inclusion criteria are as follows: (1) outpatients with SLE diagnosed at least 1 year prior to study enrolment, (2) aged 18–65 years, (3) any disease situation (active, in remission or clinically quiescent and serologically active), (5) formal consent to participate in the study.

Exclusion criteria are as follows: (1) vital organ involvement (lupus nephritis, severe neurological and/or haematological involvement), (2) SLE limited to the skin, (3) advanced chronic kidney disease (dialysis or kidney transplant); (4) important concomitant disease (cancer, diabetes, etc.); (5) mental illness and/or sensory or cognitive deficits; (6) insufficient Spanish language skills and (7) participation in another experimental study.

#### Health care professionals

Rheumatologists working in the rheumatology department of the hospitals taking part who take care of SLE patients, agree to participate in the study and remain throughout the follow-up period are included after signing informed consent.

### Setting and recruitment

Participating professionals and patients are recruited at the four public university hospitals of the Canary Islands (Spain), two of them located in Tenerife (University Hospital of the Canary Islands and University Hospital Ntra. Sra. de Candelaria) and the other two located in Gran Canaria (University Hospital of Gran Canaria Dr. Negrín and University Hospital Insular of Gran Canaria). Tenerife and Gran Canaria are the main and most populated of the Canary Islands with an approximate population of 900,000 and 840,000 people, respectively.

Rheumatologist recruitment was supported by informative meetings led by the principal researcher that included a 40–50 min presentation reporting the study objectives, planned time frame, tasks for healthcare professionals, expected resources utilization and funding procedures.

Each participating rheumatologist completed an informed consent form and agreed to consecutively invite and recruit all patients fulfilling selection criteria after providing information about the study. Recruited patients signed an informed consent form and completed the baseline questionnaires.

### Random assignment

Allocation by cluster was performed with hospitals as randomization units. After selection of professionals, an investigator blinded to hospital identity randomly assigned two hospitals to the intervention group and two hospitals to the control group by simple generation from a list of random numbers.

### Blinding

Due to the nature of the intervention, rheumatologists cannot be blinded after assignment to the intervention or control group. Participating patients from each selected hospital and the investigator responsible for data analysis will be blind to the intervention assignment until the end of the trial.

### Intervention

Participating rheumatologists assigned to the intervention group receive a multicomponent intervention designed according to the conceptual framework of behavioural change [23, 24] and person centred care model [25, 26], constituted by the following components: A) educational group programme, B) continuous support by means of an automated decision aid tool embedded into the electronic clinical record for patients included, and C) periodic feedback on process and outcome measures for all SLE patients seen. Although this set of interventions applies only to professionals, some outcome measures will be ascertained among patients included.

A) Educational and training group programme

This consists of 6 h face-to-face group training divided into two sessions, 3 months apart. Contents of the first session are designed to attain the following objectives: 1) update evidence-based clinical knowledge on SLE management according to the contents of the SLE-CPG [10] and 2) develop skills to improve communication and negotiation abilities in the context of the person-centred care model [26] and shared decision making [25]. A set of short video-films and role-playing exercises representing different types of complex sham patients are used to deliver this intervention. The aim is to train professionals to: 1) foster a climate of participation and establish clear and efficient communication; 2) elicit the patient’s concerns and preferences about possible decisions; and 3) promote shared decision making based on the best evidence, professional experience and the patient’s values and preferences in those decisions where either considerable uncertainty or several proven alternatives exists. The first part of the session is led by the clinical leader of the development of the SLE-CPG [10], a recognized rheumatologist expert in SLE, while the second part is led by two psychologists with proven expertise in patient-centred care methods and communication skills. The session also includes an explanation and training on use of the electronic decision aid tool (see below) led by the study’s principal researcher.

The second session (3 h) is designed to reinforce the skills acquired on the patient-centred care model and shared decision making [21, 22].

Both sessions are video-recorded to standardize training and to ensure intervention reliability [27].

B) Electronic decision support (EDS) system

Rheumatologists included in the intervention group have access to an EDS system built by means of a computational algorithm from the CPG-SLE [10] and integrated into the electronic clinical record to provide evidence based recommendations personalized to the specific situation and needs of every patient. The tool is made available for patients included in the study [28].

Not all CPG-SLE recommendations are included in the EDS system but only those aimed at management of patients meeting our selection criteria. These are those related to general management of the disease (monitoring, treatment, healthy lifestyle, photo protection measures and educational programmes), sexual and reproductive health, and management of pregnancy, main SLE comorbidities (cardiovascular risk, osteoporosis, infection and cancer), antiphospholipid syndrome, lupus arthritis and mild mucocutaneous manifestations. Thus, recommendations for management of other specific clinical manifestations such as lupus nephritis, haematological, neuropsychiatric and severe mucocutaneous manifestations are not included [10].

Since information on pharmacological prescription is not directly available in the electronic medical record but in a separate module, it has been necessary to develop two components of the EDS system:

- First component: on the home page of the electronic clinical record, physicians have a recommendation form at their disposal to open voluntarily. When opened, the form retrieves certain data from the patient’s clinical history and requests other information of interest, providing recommendations to support decision making, excluding treatment. During 18-month follow-up, physicians are encouraged to use this tool.
- Second component (treatment recommendations): system passively activated when entering the electronic medical prescription module (digital service that enables the physician to set and send pharmacological prescriptions by electronic means, based on information and communication technologies, which can subsequently be dispensed). Depending on the patient’s stored information and how it varies during follow-up, the system automatically displays defined recommendations about treatment adapted to the patient’s changing circumstances, providing dynamic and interactive support for clinical management of decision-making.

C) Feedback

Every three months during the 18-month follow-up, participating rheumatologists receive feedback on their clinical management of SLE patients consisting of an information sheet displaying a personalized graphical summary of relevant process indicators compared to mean results obtained by their own services. The document is received by email and graphically displays combined indicators, periodically generated by automated audit of electronic clinical records of all SLE patients (whether or not included in the study).

Process indicators displayed include: 1) prescription of antimalarials to SLE patients 2) prescription of antimalarials in particular to pregnant SLE women; and 3) prescription of glucocorticoids (prednisone, deflazacort and methylprednisolone) according to SLE-CPG recommendations. For every indicator displayed on the sheet, mean reference values obtained from all rheumatologists at the same hospital are used as dynamic comparators.

Participating rheumatologists assigned to the control group continue usual practice and do not receive any intervention.

### Outcome measures

#### Primary outcomes

The study’s primary endpoint is mean change in SLE activity self-perceived by the patient from baseline to end of follow-up using the Systemic Lupus Erythematosus Activity Questionnaire (SLAQ). This includes assessment of 24 symptoms related to the disease from 0 to 3 in accordance with severity, occurrence and severity of a flare (no flare, mild, moderate or severe) and overall lupus activity from 0 (no activity) to 10 (most activity). Total score can range from 0 to 44 [29]. SLAQ will be self-administered at baseline, and at 9 and 18 months of follow-up.

#### Secondary outcomes

##### Adherence of professionals to recommendations

Recommendations expected to have a greater impact on patients’ health which are those related to prescription of antimalarials and glucocorticoids will be analyzed. Degree of professional adherence to recommendations will be determined through data collection from clinical records at baseline and 18 months of follow-up in terms of percentage of patients with adequate treatment according to SLE-CPG recommendations. The appropriateness of treatments prescribed will be assessed and treatments will be classified as adequate (agree with antimalarials and glucocorticoids choice, dosage, dosing interval and duration of treatment) or inadequate.

##### Health-related quality of life

The following instruments are administered to patients:

- EuroQol-5D-5 L (EQ-5D-5 L)

This is a generic measure of health-related quality of life (HRQoL) comprising five dimensions: mobility, self-care, usual activities, pain/discomfort, and anxiety/depression. Each dimension is graded on five levels: no problems (level 1), slight problems, moderate problems, severe problems, and extreme problems (level 5). Health states defined by combining one level from each dimension, ranging from 11,111 (full health) to 55,555 (worst health), are converted using a scoring algorithm based on public preferences into an EQ-5D-5 L single index value, ranging from 1 (perfect health) to 0 (health state equivalent to death). Negative values represent health states considered to be worse than death [30]. HRQoL will also be self-administered at baseline, 9 and 18 months. The questionnaire also includes a visual analogue scale (EQ-VAS) where responders are asked to indicate their health status on the day of the interview, ranging from 0 (worst possible health) to 100 (best possible health).

- Systemic Lupus Erythematosus Quality of Life questionnaire (LupusQol)

This is a 34-item disease-specific scale for measuring HRQoL in adults with SLE. Eight domains are covered, including physical health, emotional health, body image, pain, planning, fatigue, intimate relationships, and burden to others. LupusQol has a 5-point Likert response format (all the time, most of the time, a good portion of the time, occasionally and never). The mean raw domain score is transformed to scores ranging from 0 (worst HRQoL) to 100 (best HRQoL) by dividing by 4 and then multiplying by 100. The result represents the transformed score for that domain. The mean raw domain score is then calculated by totalling the item response scores of the answered items and dividing by the number of answered items. LupusQol will be administered at baseline and at 18 months [31].

##### Patient perception of their participation in decision making

Assessed by the Shared Decision-Making questionnaire (SDM-Q-9). This 9-item instrument measures the extent to which patients are involved in the process of decision-making from the patient’s perspective. Each item is rated on a 6-point Likert scale (0–5). Scores range from 0 to 45. Multiplication of the raw score by 20/9 provides a score transformed to range from 0 to 100, where 0 indicates the lowest possible level of shared decision-making and 100 indicates the highest extent of shared decision-making. SDM-Q-9 will be administered at baseline, at the end of every subsequent visit within the follow-up period, and at 18 months [32, 33].

##### Attitude of professionals to partnership with the patient for shared decision making

Assessed by the Leeds Attitude Towards Concordance II Scale (LATCon II). This is a 20-item scale for measuring health care professionals’ attitudes toward concordance in medicine taking. Each item is rated on a 4-point Likert scale (0–3). Scores on the LATCon II range from 0 to 60, with higher scores representing more positive attitude towards concordance [31, 32]. LATCon II will be administered to rheumatologists in the intervention group at baseline, before and after the educational group programme, and at 18 months.

##### Knowledge of professionals about SLE management

Knowledge level of recommendations for SLE management formulated in the SLE-CPG [10] will be assessed by mean of a 10-question test developed ad hoc administered to rheumatologists. Responses to questions scored 1 point if correct or 0 point if incorrect. Thus, possible scores ranged from 0 to 10. This instrument will only be administered to rheumatologists of the intervention group before and after the educational group programme, and at 18 months.

##### Health care utilization and productivity losses

Costs due to resources used alongside the clinical management of SLE patients in intervention and control groups will be assessed from the healthcare services perspective, including costs related to development, implementation and use of all components of the intervention assessed (group sessions, computer-assisted aid and feedback).

Information about prescribed medications and doses, patient contacts with primary care services, outpatient visits, hospital admissions and length of stay, emergency attendances as well as travel and productivity losses will be collected using patient questionnaires at 9 and 18 months.

#### Additional measures

Sociodemographic data will be collected at baseline from patients and rheumatologists. Professionals will be asked about their age, sex and professional profile (years in practice, degree of expertise in SLE, etc.), while patients will be asked about their sex, age, education level, occupation, employment situation, marital status, family living status (alone or accompanied), and age at SLE diagnosis.

### Data management

Data collection is managed by means of an electronic case report form designed for this study and complemented with patient questionnaires self-completed at baseline, and at 9 and 18 months by phone. Information from professionals is obtained by means of self-reported questionnaires.

On receipt of their consent forms, all participants (professional and patient) is assigned a code which will link their data to a master-sheet which will contain participants’ demographic details and location. The mastersheet will be kept secure and confidential by the project coordinator in a password-protected file in the Project data management system. All data for the evaluation of the project will be identified by the participants’ code only. This is so that the data can be kept confidential but can be re-identified to enable appropriate grouping and analysis in the case studies at the end of the study.

Hard copies of all consent forms and questionnaires will be stored in locked filing cabinets in the Evaluation Service of the Canary Health Service (or password-protected folder if in soft copy form) up to 5 years after finishing the trial.

The planning of information collection for every outcome measured throughout the project is shown in Table 1.

**Table 1:** Outcome measurements according to time points of collection

Time point	Outcomes measured on patients	Outcomes measured on rheumatologists
T1 (baseline)^a^	Demographic data SLAQ [29]; EQ-5D-5L [30]; LupusQoL [31]; SDM-Q-9^c^ [32]	Demographic data; professional profile; Adherence to recommendations; LatConII^d^ [34, 35]; Knowledge test^d^
T2 (9 mo)^b^	SLAQ [29]; Health care utilization and productivity losses	
T3 (18 mo)^b^	SLAQ [29]; EQ-5D-5L [30]; LupusQoL [31]; Health care utilization and productivity losses	Adherence to recommendations; LatConII [34, 35]; Knowledge test

*Abbreviations*: *LatConII* Leeds Attitude Towards Concordance II Scale, *LupusQoL* Systemic Lupus Erythematosus Quality of Life questionnaire, *SDM-Q-9* Shared Decision-Making questionnaire, *SLAQ* Systemic Lupus Erythematosus Activity Questionnaire, *mo* months

^a^Self-reported face to face

^b^Self-reported by telephone

^c^Also after each consultation during study follow-up

^d^Before and after the educational group programme

### Statistical methods

The main analysis for primary and secondary endpoints will be based on multilevel mixed models, including as covariates the baseline value of the dependent variable, the severity of the patient and the professional’s level of baseline knowledge. First level variables will be those corresponding to each measurement throughout follow-up (related time measurements), second level includes patient variables and third level variables correspond to hospital (cluster). The effect that identifies the intervention arm will be considered fixed for the different hospitals, whilst the intercept will be considered random. The model will also include an interaction term between arm and month, allowing for differences in the intervention effect between follow-up assessments [36]. The adjusted estimated mean will be calculated for each moment of follow up compared to baseline. To accommodate missing values in the effect analyses, the multiple imputation procedure in STATA V15.0 will be used [37]. This procedure saves cases for analysis and can be considered as an intention-to-treat analysis. Differences will be considered statistically significant if *p*-value < 0.05.

### Sample size calculation

A total of 418 patients and 20 professionals (5 per hospital) will be required to detect an effect size of 0.3 (small) in the activity of the SLE (SLAQ) assuming a standard deviation of 8 and an intra-class correlation coefficient (ICC) of 0.01 with a power of 80% and a level of statistical significance of 5%.

### Economic evaluation

Cost-effectiveness evaluation of the multicomponent intervention will adopt the twofold of the SHS and social perspective. Effectiveness of the intervention will be measured by means of results of the SLAQ scale, along with Quality Adjusted Life Years (QALY) provided by the EQ-5D-5 L. The costs of the two comparative strategies will be calculated using the average cost estimated based on use of health resources obtained from patients during 18-month follow-up of the RCT. To allow cost-effectiveness estimation from a social perspective, information on indirect costs (travel and productivity losses) will be collected at 9 months and 18 months of follow-up. Unit costs will be taken from standard published sources when available and from specific providers. The incremental cost-effectiveness ratio (ICER) defined as the difference in average costs of each alternative (multicomponent vs. usual) divided by the difference in the average effectiveness of each alternative will be estimated. This will enable calculating the incremental cost per unit of effectiveness (SLAQ and QALY) gained from the intervention under study. Nonparametric methods will be used to calculate the confidence intervals on the ICER using the bootstrapping analysis, which will also be used to calculate the cost-effectiveness acceptability curve that shows the probability that an intervention will be cost-effective for different values of the SHS availability to pay for an additional unit of effectiveness. We will also conduct extensive deterministic and probabilistic sensitivity analyses.

### Duration of fieldwork

Fieldwork is estimated to last 3 years. The first year to complete recruitment of patients and rheumatologists and the following 2 years for follow-up and measurement. As interventions are maintained over time, the period of intervention and follow-up overlap (Fig. 1).

### Monitoring

Trial monitoring is the responsibility of a research team in charge of all quality control activities, assessing adherence to the trial protocol, timely work plan execution and comprehensiveness of data acquisition and data quality (databases have been designed to avoid downloading inappropriate values for every variable).

### Trial status

The trial is ongoing with patient recruitment completed June 302,019. The study is in the intervention and data collection stages at the time of the protocol manuscript submission.

## Discussion

The ongoing study is a two-arm cluster randomized controlled trial assessing the comparative effectiveness and cost-effectiveness of usual implementation of a CPG guideline to manage SLE patients against a multicomponent intervention for implementation of such CPG. The intervention combines conventional group educational and training activities promoting patient-centred care and shared decision making between patients and clinicians [25, 26], an EDS system according to SLE-CPG recommendations, and periodic feedback on their longitudinal clinical performance. Primary analysis is aimed at comparing the mean 18-month SLAQ score change from baseline among SLE patients attending hospitals assigned to the control group with the mean 18-month SLAQ score change among SLE patients attending hospitals assigned to the intervention group. Cluster randomization is used to reduce the risk of contamination bias, since the educational component of the intervention was applied to groups.

The results of this study are expected to generate scientifically valid information relevant for clinicians, managers and health policy makers; adding clinical and economic evidence to the scientific field of knowledge transfer and behaviour modification. To date, there are still few studies that assess whether implementation strategies targeting the use of CPGs are effective in patient outcomes. Even less attention has been paid to the costs of those implementation strategies despiste some authors heve revealed the importance of economic evaluation in this context [38].

Response rate of patients and difficulty obtaining all the data required are expected to be the main problems of this study. To reduce the risks of low response rates and high losses to follow-up, major effort has been made to explain to patients the objectives of the study at enrolment and during follow-up visits. Questionnaires will be completed over the phone and the option of sending these to patients by mail is always offered.

This study is not free from limitations. Firstly, the follow-up of 18 months may be insufficient to observe relevant changes in disease activity (health outcome variable), since the SLE is a disease that occurs with outbreaks. For this reason, intermediate variables (process) that measure adherence to CPG-SLE recommendations will be obtained. Secondly, both the measurement of effects and cost sharing of the intervention evaluated in this proposal will be limited to rheumatologists; professionals from other specialties involved in the care of these patients are left out of the intervention. Therefore, it is possible that both measures of effectiveness and cost-effectiveness offer conservative results (smaller effect size).

## Data Availability

The datasets generated and/or analyzed during the current study are not publicly available for confidentiality reasons but are available from the corresponding author on reasonable request. Once the dataset has been completed, it will be available at clinicaltrials.gov Identifier: NCT03537638.

## Abbreviations

CPG: Clinical practice guideline
CPG-SLE: Clinical practice guideline for the clinical care of systemic lupus erythematosus
EDS: Electronic decision support
EQ-5D-5 L: The 5-level EQ-5D version
GEE: Generalized linear model
HRQoL: Health-related quality of life
ICC: Intra-class correlation coefficient
ICER: Incremental cost-effectiveness ratio
LATCon II: Leeds Attitude Towards Concordance II Scale
LupusQol: Systemic Lupus Erythematosus Quality of Life questionnaire
QALY: Quality-adjusted life years
SDM-Q-9: Shared Decision-Making questionnaire
SHS: Spanish Healthcare System
SLAQ: Systemic Lupus Erythematosus Activity Questionnaire
SLE: Systemic lupus erythematosus
